# *Bos indicus* Carcasses Suspended by the Pelvic Bone Require a Shorter Aging Time to Meet Consumer Expectations Regarding Meat Quality

**DOI:** 10.3390/foods12050930

**Published:** 2023-02-22

**Authors:** Welder Baldassini, Marcelo Coutinho, Gregori Rovadoscki, Bruna Menezes, Murilo Tagiariolli, Juliana Torrecilhas, Júlia Leonel, Guilherme Pereira, Rogério Curi, Otávio Machado Neto, Luis Artur Chardulo

**Affiliations:** 1 Animal Nutrition and Breeding Department, School of Veterinary Medicine and Animal Science (FMVZ), São Paulo State University (UNESP), Botucatu 18610-034, SP, Brazil; 2 Animal Science Department, School of Agriculture and Veterinary Science (FCAV), São Paulo State University (UNESP), Jaboticabal 14884-900, SP, Brazil; 3 Research and Development Department, Brazil Beef Quality Ltd., Piracicaba 13415-000, SP, Brazil

**Keywords:** beef cattle, sensory, shear force, tenderness, zebu

## Abstract

This study evaluated the effects of hanging the carcass by the Achilles tendon (AS) versus pelvic suspension (PS) on meat quality traits. *Bos indicus* carcasses of two distinct biological types/sex categories comprised 10 young Brangus heifers and 10 Nellore bulls which were finished in a feedlot. Half-carcasses of each biological type/sex category were randomly hung using Achilles suspension (*n* = 20, AS) or pelvic suspension (*n* = 20, PS) for 48 h. At boning, longissimus samples were collected for evaluation by untrained consumers for tenderness, liking of flavor, juiciness and overall acceptability, after aging for 5 or 15 days. Objective samples were also tested for shear force (SF), Minolta meat colour, ultimate pH, cooking loss (CL) and purge loss (PL). There was a positive effect (*p* < 0.01) of PS on the sensory tenderness of Nellore bulls and Brangus heifers aged for 5 days compared to the AS method. At 15 days of aging, difference in sensory tenderness was observed (*p* < 0.05) in either group. Additionally, an interaction occurred between the suspension method and the aging of Nellore beef (*p* < 0.05) on liking of flavor, juiciness and overall acceptance, while the same effects were not observed for Brangus beef (*p* > 0.05). Nellore carcasses submitted to PS tended (*p* = 0.06) to produce more tender meat than those submitted to AS (SF = 44.62 ± 6.96 vs. 50.41 ± 8.04 N), and lower CL (*p* < 0.05) were found (27.7 vs. 30.9%). Carcass-suspension methods did not influence meat color, pH or PL in either group (*p* > 0.05). The PS contributes to improve the quality of *Bos indicus* bulls loins; in addition, this method allows a reduction in the aging time from 15 to 5 days, and it can be used to supply meat consumer markets which accept a certain level of eating quality.

## 1. Introduction

The beef cattle herd in Brazil is composed mainly of Zebu animals (*Bos indicus*), with a predominance (roughly 80%) of the Nellore genotype. Despite their adaptation to tropical climate, Nellore breed take longer to reach targeted sales weight compared to taurine cattle (*Bos taurus*), such as Aberdeen Angus and Hereford. Many farms in the southeast and midwest regions rear Nellore animals on pasture, followed by finishing in the feedlot, a system in which the use of bulls is common [1]. This biological model of production supplies a large part of the domestic and export market [2]. It is well known that the meat quality of *Bos indicus* bulls is limited, especially in terms of tenderness [3,4], when compared to *Bos taurus* and crossbred animals, with the need for a longer aging time to improve this trait. However, considering the costs of a meat-packing plant, very long aging times can render the product excessively expensive.

The use of crossbred cattle (*Bos taurus* × *Bos indicus*) finished in the feedlot and slaughtered at a young age has been adopted as a strategy to improve productive performance and meat quality [5], especially tenderness, within a shorter post-mortem aging period. Additionally, crossbred castrated male and female cattle [6] are increasingly used since there are consumers who are willing to pay more for higher-quality meat products [7]. It is known that the gender of cattle affects animal performances and growth, and heifer have superior carcass fatness and meat quality traits compared to bulls, as reported by [8,9]. To supply their own butcher shops, some Brazilian meat-packing companies pay better for carcasses derived from this biological type (low *Bos indicus* content, heifers or steers carcasses feedlot-finished and slaughtered at a young age).

In the meat-packing industry, the commonly used hanging method of bovine carcasses is suspension by the Achilles tendon (AS). This method results in the shortening of selected hindquarter muscles during rigor mortis, which can affect meat quality. On the other hand, suspension of the carcass by the pelvic bone, a method known as tenderstretch or PS, is widely used by the Australian and European meat-packing industry in order to improve sensory meat quality, particularly tenderness, as well as reduce the variability of this trait in some muscles [10]. As described by Ferguson [11], the PS method does not affect the semitendinosus, as this muscle is already stretched and therefore shows no improvement in eating quality. During the post-mortem chilling period of carcasses, the tenderness of almost all hindquarter muscles can be improved by the PS method. Studies suggest that this method may be more effective for tougher meats [12] and may be an alternative to improve the meat quality of bulls [13]. In the Meat Standard Australia (MSA) beef-grading program [14], there is no interaction between *Bos indicus* and carcass-suspension method on the prediction of eating quality.

Carcass suspension by the pelvic bone is a technique that is inexpensive to implement, producing gains in meat quality [15,16] and adding value to meat products. In Brazil, studies evaluating the effects of PS on meat quality are scarce. Furthermore, there are no studies of the effects of PS on the meat of *Bos indicus* and crossbred cattle, evaluating sensory and objective traits in different sex classes and under different aging conditions.

Within this context, meat from carcasses suspended by the pelvic bone may require a shorter aging time to meet consumer expectations regarding quality traits, especially beef of carcasses of males with a high *Bos indicus* content. Only a few studies with Nellore bulls and *Bos indicus* carcasses described the interaction of the carcass-suspension method and meat aging, which allows the investigation of its effects on meat quality traits (sensory and objective variables). Therefore, the aim of this study was to evaluate whether PS can improve quality traits of aged meat from crossbred heifers and Nellore bulls, two contrasting biological types that supply different meat consumer markets in Brazil and abroad.

## 2. Materials and Methods

The study protocol was approved by Ethics Committee (CEUA) of the São Paulo State University “Júlio de Mesquita Filho”–UNESP, Campus of Botucatu (protocol number 0094/2020; approved date: 12 August 2020).

### 2.1. Animals and Carcasses

Brangus heifers (*n* = 10) and Nellore bulls (*n* = 10), finished in commercial feedlots (Boituva, São Paulo, Brazil), were evaluated. The animals belonged to the same management group and were submitted to the same nutritional conditions and feedlot period. The two groups received the same diet, containing 15% forage (sugarcane bagasse) and 85% corn-based concentrate (soybean meal, ground corn, urea, mineral mixture). The animals were allocated in collective pens, equipped with a bunk and an automatic drinking trough. The diet was offered *ad libitum*, twice a day at 8 a.m. and 4 p.m. The experimental groups were slaughtered at different times (Brangus heifers = 100 days on feed; Nellore bulls = 120 days on feed). The slaughter weight of the animals of each group was established by the commercial protocol of the slaughterhouse (Boituva, São Paulo, Brazil) based on the standardization of their meat products. At the end of feeding period, the final body weight (FBW) was record for Brangus heifers (FBW = 510 ± 41 kg) and Nellore bulls (FBW = 590 ± 57 kg). The aim of using two such distinct groups was to evaluate the main biological types destined for the two most important models of Brazilian meat production. Thus, Nellore animals were classified as products of the commodity model—domestic and export market—and crossbred Brangus animals as the premium quality model.

During processing of the two slaughters, the number of permanent incisors teeth were verified and carcasses were cut longitudinally into two parts and weighed individually. Carcasses were not electrically stimulated in the current study. The half-carcasses were randomly suspended by the traditional method (AS; *n* = 20: 10 Brangus and 10 Nellore) or by the tenderstretch method (PS; *n* = 20: 10 Brangus and 10 Nellore). After identification, the carcasses were kept in the cold room (1 °C) for 48 h.

Next, the carcasses were evaluated in the chiller, and the following data were collected: carcass weight; ossification score (physiological maturity); hump height (*Bos indicus* percentage); fatness score; conformation score; marbling score; backfat thickness; ribeye area; and ultimate pH (48 h) [17]. Briefly, ossification was assessed by one trained technician, which scored the degree of ossification of the vertebral spinous processes as well as the shape and colour of the rib bones, with the lowest score being 100 and the highest being 590. The hump height was recorded in gradients of 5 mm, measuring all of the meat across the hump from the line formed by the dorsal ends of the spinous processes and extended cranially along the dorsal edge of the *Ligamentum nuchae*, across to the dorsal surface of the *Rhomboideus* muscle.

Additionally, fatness score was assessed, using 1 to 5 degrees for the distribution of fat cover in the hot carcass (1 = absent; 2 = scarce; 3 = medium; 4 = uniform; 5 = excessive). Subsequently, conformation score was assessed by development of muscle mass in the hindquarters region (1 = concave; 2 = sub-straight; 3 = straight; 4 = sub-convex; 5 = convex). Finally, marbling score was assessed using MSA standard photograph, divided into tenths for grading, creating a score range from 100 to 1100 in increments of 10. Backfat thickness (BFT, mm) and ribeye area (REA, cm^2^) were measured at the 12th/13th rib interface.

After deboning, *Longissimus thoracis et lumborum* (LTL) samples (~12 cm) were collected from each half-carcass (AS and PS; two samples each) between the 11th and 13th rib (cranial direction) for subsequent sensory and laboratory analyses of meat quality.

### 2.2. Aging

All meat samples collected during deboning from treatments AS and PS were submitted to wet aging for 5 and 15 days at a temperature of 0 to 2 °C in a biological oxygen demand (BOD) incubator (TE-371, TECNAL, Piracicaba, São Paulo, Brazil). Before aging, the samples were cut into 2.54 cm-thick steaks, vacuum packed, and duly identified with the treatment (AS or PS) and aging time (5 or 15 days). The position of the steak for aging periods was fixed. After the aging period, the steaks were packed separately in plastic bags for vacuum sealing with low oxygen permeability and stored frozen at −20 °C until the time of analysis. Two steaks per treatment and aging time were used in the sensory tests and the other two steaks were used for the objective analysis of meat quality, as described below.

### 2.3. Sensory Analysis

For sensory analysis, untrained consumers assigned scores from 0 to 100 on unstructured hedonic scales to the following meat quality traits using the Sensory Meat software v.1.6 (Brazil Beef Quality Ltd., Piracicaba, São Paulo, Brazil): tenderness, liking of flavor, juiciness, and overall acceptance, which was based on MSA consumer testing, as described [18]. The length of the lines in the scoring sheets used in this experiment were 100 mm. For participation, the consumers were determined to be between 18 and 70 years old, to consume meat at least once a week, and to prefer meat medium-rare. For this study, the genetic groups (biological types) were evaluated separately.

Forty-eight consumers were used in each experiment, with two sessions comprising 24 consumers divided into two groups (G1 and G2) of twelve consumers each. G2 was a repetition of G1 to increase the error variance estimate. The consumers received seven samples, with the first tasting being used as the reference sample (standardized benchmark). For this purpose, one of the tested samples was selected randomly in each session. After they had tasted the reference samples, the consumers analyzed the six subsequent samples from three animals, with each animal being submitted to the two suspension methods, left side of the carcass (PS) and right side (AS), corresponding to the six samples.

The LTL samples were thawed for 24 h at 5 °C in a refrigerator (Gelopar, GREP-4PAI, São Paulo, Brazil) and had a temperature less than 10 °C before preparation. The steaks (2.54 cm thick) were immersed in saline (5% NaCl), and then grilled on a gas grill (Char-Broil Signature 3Q—Infrared, Columbus, GA, USA). The internal temperature was monitored with thermocouples (ThermoPro, Model TP-16, Hong Kong, China). The steaks were removed from the grill when their geometric center reached a temperature of 63 °C. After preparation, the steaks were cut into subsamples (1.27 cm × 1.27 cm × 2.5 cm), packed in sealed pots for the maintenance of humidity, and stored in stoves for temperature control and stabilization, before being served to the consumers. The samples were served in single-use small boxes (Brazil Beef Quality Ltd., Piracicaba, São Paulo, Brazil) with divisions to prevent visual comparison between samples, and to maintain the temperature during assessment (Figure S1).

### 2.4. Laboratory Analysis

Beef samples from the two treatments (aged for 5 and 15 days) were used to analyze meat color, pH, shear force (SF), cooking loss (CL) and purge loss (PL) at the Meat Science Laboratory of the São Paulo State University (UNESP Botucatu, São Paulo, Brazil).

#### 2.4.1. pH and Meat Color

To allow blooming time, the beef samples were thawed at 4 °C for 24 h and exposed to oxygen for 30 min at 4 °C. Initially, the meat pH was measured using a digital pH meter equipped with a penetration probe (Model AK86, AKSO Instruments, Rio Grande do Sul, Brazil). Standard buffers (pH 4.0 and 7.0) were used in calibration procedures. In the same steak, meat color (L * = lightness, a * = redness, b * = yellowness) was measured using the CIELab system of the CR-400 colorimeter (light source A, observer angle 10°, aperture size 5.0 cm, and display Y: 0.01 at 160.00% reflectance; Konica Minolta Sensing, Inc., Tokyo, Japan), following previously described procedures [19]. Briefly, the colorimeter was calibrated using a standard black and white plate and three-color readings were then performed on the surface of the LTL muscle sample at room temperature (~20 °C). The mean of these measurements was then calculated. The chroma colorimetric index was calculated using the formula $\sqrt{{\left(a*\right)}^{2}+{\left(b*\right)}^{2}}$, and hue was calculated using $tan{\left(\frac{b*}{a*}\right)}^{-1}$, as described [20].

#### 2.4.2. Purge Loss, Shear Force and Cooking Loss

One steak from each treatment was used to determine PL, as well as SF and CL at 71 °C, considering the two aging times (5 or 15 days). The recommendations of the American Meat Science Association were followed [21]. The PL of the beef loin sections was determined by measuring the difference between the initial weight prior to freezing and the final weight after freezing/thawing. Thus, the PL of never frozen control beef loins was not assessed. The CL was divided into evaporation loss (EL) and drip loss (DL), determined as percentage. The DL was obtained by weighing only the refractory before and after cooking the sample. The EL was obtained by weighing the sample before and after cooking.

The beef loins were weighed and placed in a grid over a glass refractory. Next, a thermometer (Model 612 digital thermometer, ATP Instrumentation, Ashby-de-la-Zouch, England) was inserted into the geometric center of the samples to monitor the internal temperature. Samples were cooked in a preheated oven (Feri90 Venâncio Aires, Rio Grande do Sul, Brazil). At 40 °C, the beef samples was turned and remained in the oven until an internal temperature of 71 °C was reached. The samples were then kept at 21 °C for 15 min and weighed.

Grilled steaks were chilled for 24 h at 4 °C. After this period, cylinders (12.7 mm) were cut from the sample center in the longitudinal direction towards the muscle fiber using a nozzle coupled to an industrial drill. From the total of grilled steaks, at least six to eight technical replicates (cylinders) from the same half carcass (LTL sample) were sheared perpendicular to the fiber direction, as recommended by Wheeler et al. [22]. A Brookfield CT-3 Texture Analyzer (AMETEK Brookfield, Middleborough, MA, United States), equipped with a stainless steel 3.07 mm-thick Warner–Bratzler blade with a V-shaped (60° angle) cutting edge was used, and the SF results were reported in Newton (N).

### 2.5. Statistical Analysis

The data set of meat quality traits’ two biological types were analyzed separately. The sensory test sessions were organized considering two block factors, consumer and assessment order, in a neighbor-balanced Latin square design (6 × 6) [23,24]. A mixed generalized linear model was applied considering a binomial distribution of the data, using the PROC GLIMMIX procedure (SAS v.9.4). The length of the lines in the scoring sheets used in this experiment were 100 mm. Clipping procedures to reduce standard error of the sensory mean was adopted, as recommended by [18]. In brief, for each session within each sample, the two highest and the two lowest values for each trait evaluated in sensory analysis were eliminated.

For the response variables tenderness, liking of flavor, juiciness and overall acceptance, the model included the sequence (sample tasting), suspension method (AS and PS) and aging time (5 and 15 days) as fixed effects and the consumer as random effect. Tukey’s test was used for the comparison of means (*p* ≤ 0.05). Interaction terms for suspension method × aging were tested as follows:

Y = μ + C + S + T + A + T × A + e

where Y = trait of interest (sensory meat quality trait); μ = overall mean; C = effect of consumer; S = effect of sequence; T = effect of carcass-suspension method; A = effect of aging; T × A = effect of the interaction; and e = random error.

For laboratory variables, the model included the suspension method (AS and PS) and aging time (5 and 15 days) as fixed effects. Interaction terms for suspension method × aging were also tested, according to the model:

Y = μ + T + A + T × A + e

where Y = trait of interest (objective meat quality trait); μ = overall mean; T = effect of carcass-suspension method; A = effect of aging; T × A = effect of the interaction; and e = random error.

In addition, the data were evaluated by Pearson correlations. Subsequently, the data were evaluated by principal component analysis (PCA) using the R software (v. 4.1.2) to achieve an overview of their correlations and to visualize and determine the degree of the contribution of suspension method to the variation in quality traits in each biological type. The FactoMineR [25] and Factoextra [26] packages were used to generate the PCA plots and graphs, respectively.

## 3. Results

### 3.1. Carcass Traits

Table 1 shows the carcass traits of the genetic/gender groups used in the present study. Both factors (genetic and gender) were used as biological model for application of different carcass-suspension methods. As expected, these factors affect *(p* < 0.01) hot carcass weight, fatness, marbling score, age (by dentition) and *Bos indicus* content (measured by hump height). The description of such data was aimed at characterizing the biological models, which supply different consumer markets in Brazil.

Fatness score was assessed using 1 to 5 degrees for the distribution of fat cover in the hot carcass (1 = absent; 2 = scarce; 3 = medium; 4 = uniform; 5 = excessive). Conformation score was assessed by development of muscle mass in the hindquarters region (1 = concave; 2 = sub-straight; 3 = straight; 4 = sub-convex; 5 = convex). Marbling score was assessed using MSA standard photograph, divided into tenths for grading, creating a score range from 100 to 1100 in increments of 10. Ossification was assessed by the degree of ossification of the vertebral spinous processes, as well as the shape and colour of the rib bones, with the lowest score being 100 and the highest being 590.

### 3.2. Meat Quality Traits

Evaluation of the sensory and physical–chemical characteristics of meat quality revealed no significant interaction (*p* > 0.05) between two factors: carcass-suspension method and genetic/gender groups. Thus, the results are reported separately as the single effect of each factor. The effects of the carcass-suspension method on the sensory and objective traits of Nellore bulls (Table 2) and Brangus heifers (Table 3) were consistent for some variables but divergent for others, considering the two aging periods studied. For both biological types, there was a positive effect (*p* < 0.01) of PS on the sensory tenderness of meat aged for 5 days when compared to the AS method. Consumers attributed higher tenderness scores to meat of the PS treatment, regardless of gender or genetic group.

When meat was aged for 15 days, there was no difference in sensory tenderness between the AS and PS methods (*p* > 0.05) in either group, indicating that a longer aging period compensated for the difference in meat quality caused by the carcass-suspension method. Additionally, there was an effect of the interaction between suspension method and aging of Nellore beef (*p* < 0.05) on liking of flavor, juiciness and overall acceptance (Table 2), while the same were not observed for Brangus beef (*p* > 0.05) (Table 3).

In the Nellore bulls group, carcasses submitted to PS tended (*p* = 0.06) to produce more tender meat (lower SF average values) than those submitted to the AS method (Table 2). Such effects were not observed for Brangus beef (*p* > 0.05), which indicates that PS method is more effective in improving meat quality traits of *Bos indicus* carcasses of bulls compared to crossbred (*Bos taurus* × *Bos indicus*) heifers. In addition, lower CL (*p* < 0.05) were found in the Nellore group whose carcasses were submitted to PS treatment compared to AS (27.7 vs. 30.9%). The carcass-suspension method and aging period (5 vs. 15 days) did not influence L *, a *, b *, chroma, hue, meat pH and PL, in either group (*p* > 0.05).

### 3.3. Correlations

**Figure 1:**
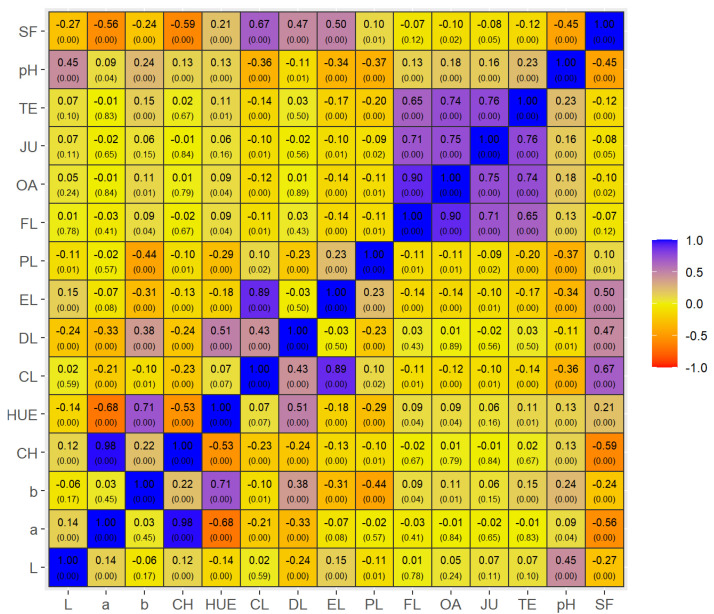
Pearson correlation (uppercase numbers) and significance (lowercase numbers; *p*-values) between meat quality traits of Nellore bulls. Blue or purple colors means strong or medium positive correlations, while orange or yellow/browned colors means strong or medium negative correlations. Variables: SF = shear force; pH = ultimate pH (48 h post-mortem); TE = sensory tenderness; JU = juiciness; OA = overall acceptance; FL = liking of flavor; PL = purge loss; EL = evaporation loss; DL = drip loss; CL = total cooking losses; HUE = real color; CH = chromaticity; b = yellowness; a = redness; and L = lightness.

**Figure 2:**
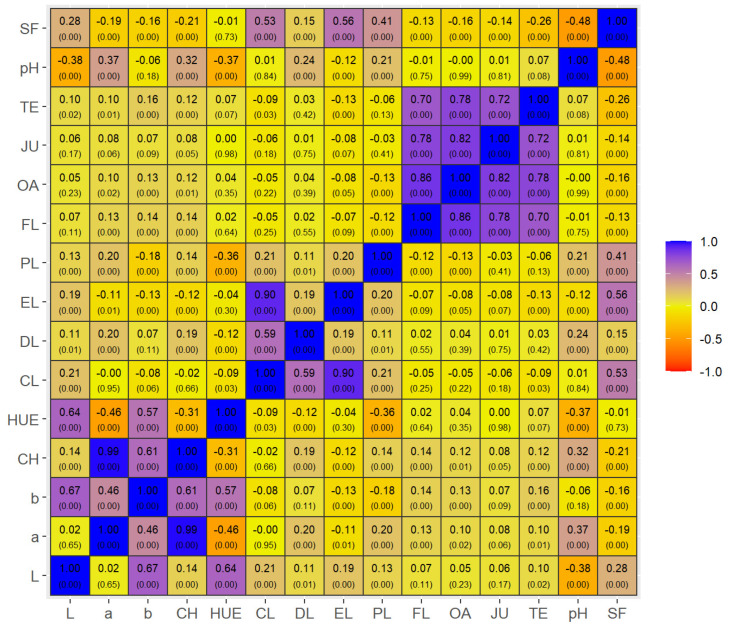
Pearson correlation (uppercase numbers) and significance (lowercase numbers; *p*-values) of Brangus heifers. Blue or purple colors means strong or medium positive correlations, while orange or yellow/browned colors means strong or medium negative correlations. Variables: SF = shear force; pH = ultimate pH (48 h post-mortem); TE = sensory tenderness; JU = juiciness; OA = overall acceptance; FL = liking of flavor; PL = purge loss; EL = evaporation loss; DL = drip loss; CL = total cooking losses; HUE = real color; CH = chromaticity; b * = yellowness; a * = redness; and L * = lightness.

The phenotypic correlations between the sensory and objective meat quality traits found in this study are considered to be of low magnitude in both Nellore bulls (Figure 1) and Brangus heifers (Figure 2). On the other hand, a strong association between the sensory traits (r ≥ 0.70; *p* < 0.01) was observed for the two groups (Nellore and Brangus) and carcass-suspension methods (PS and AS), indicating that most consumers who attributed high scores to tenderness also attributed high scores to liking of flavor, juiciness and overall acceptance.

Although of low magnitude, the correlation between sensory tenderness and SF was inverse (*p* < 0.01) for both Nellore (r = −0.12) and Brangus meat (r = −0.26). Additionally, SF was associated with higher CL in these two groups (r = 0.67 and 0.53, respectively), in agreement with the results of sensory analysis. The negative correlations between objective meat quality variables in Nellore and Brangus animals should also be highlighted, such as between SF and pH (r = −0.45 and −0.48, respectively; *p* < 0.01) and between SF and chroma (r = −0.59 and −0.21, respectively; *p* < 0.01).

**Figure 3:**
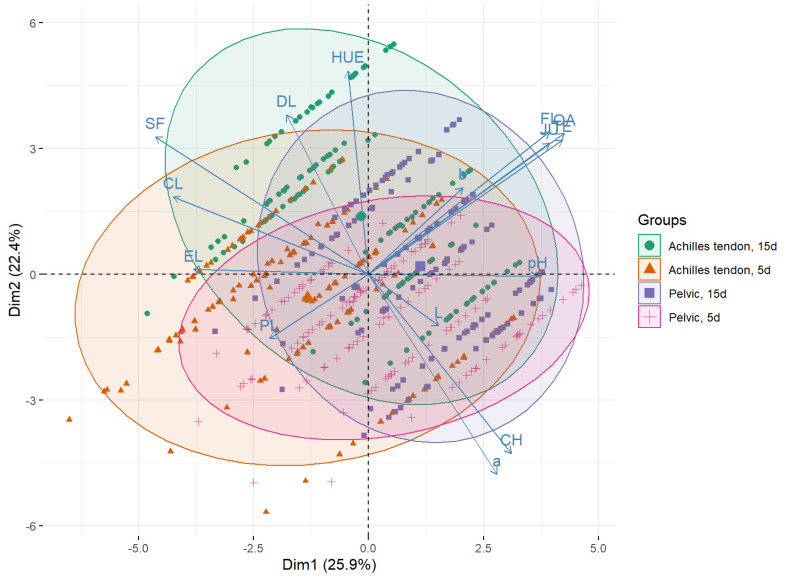
Score plot of meat quality data of Nellore bulls in the multivariate space defined by principal component analysis. PC1 “tenderness” (explained variance: 25.9%) × PC2 “meat color” (explained variance: 22.4%). Group symbols represent both the carcass-suspension method and meat aging period, respectively: ● (Achilles tendon; 15 days), ▲ (Achilles tendon; 5 days), ■ (Pelvic, 15 days), + (Pelvic, 5 days). The centroid value of each treatment is represented by a larger shape size.

**Figure 4:**
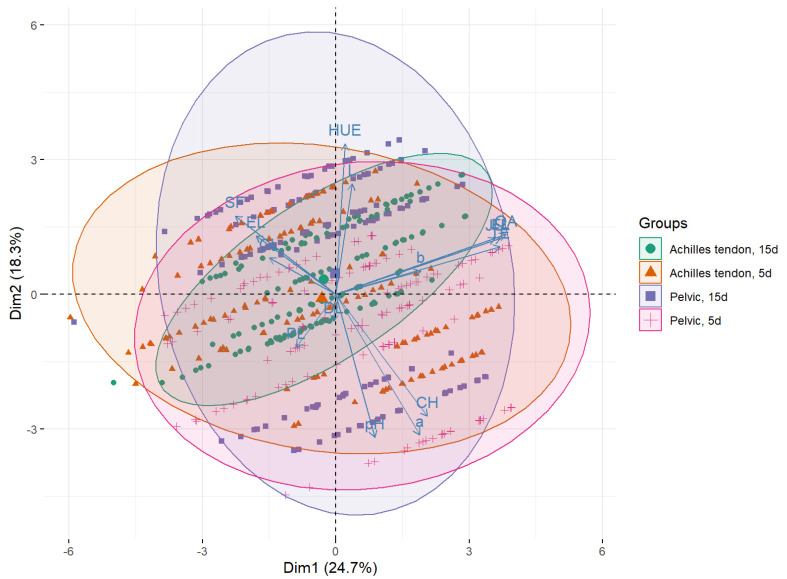
Score plot of meat quality data of Brangus heifers in the multivariate space defined by principal component analysis. PC1 “tenderness” (explained variance: 24.7%) × PC2 “meat color” (explained variance: 18.3%). Group symbols represent both the carcass-suspension method and meat aging period, respectively: ● (Achilles tendon; 15 days), ▲ (Achilles tendon; 5 days), ■ (Pelvic, 15 days) and + (Pelvic, 5 days). The centroid value of each treatment is represented by a larger shape size.

The meat quality traits (*n* = 15) were also submitted to multivariate analysis by PCA. Considering an eigenvector ≥1 as a criterion [27], the first five principle components were necessary to explain more than 80% of the total variance of the data in both groups (Table S1). In Nellore bulls (Figure 3), the first two principal components (PC1 and PC2) explained more than 48% of the total variance of the data, with the combination of palatability (sensory) variables with objective tenderness (SF and CL) and meat color (hue) being the most effective in defining PC1 and PC2, respectively (Table S2). In Brangus heifers, the proportion of variance in the data explained by PC1 and PC2 was higher than 24.7% and 18.3%, respectively (Figure 4). In this group, the variables related to palatability (sensory tenderness, liking of flavor, juiciness and overall acceptance) and meat color (hue) combined with pH were more effective in defining PC1 and PC2 (Table S3).

The biplots of the meat quality data of Nellore bulls (Figure 3) and Brangus heifers (Figure 4) show correlations of the variables with PC1 and PC2. Meat color (chroma and a *) was positively associated with PC1. The same variables were negatively (i.e., inversely, ~180°) associated with SF and CL. Thus, a high chroma value was observed in more tender meat (lower SF) and meat with a higher water-holding capacity (lower CL). Although not measured directly, CL and PL can be used as indirect measures of water-holding capacity and were negatively associated with PC2. Thus, samples with high CL were possibly derived from animals with low meat lightness (L) scores.

The PCA also revealed a better clustering/separation of the data of Nellore carcasses submitted to PS and aged for 5 days. These results reinforce the finding that the effects of PS on *Bos indicus* carcasses were more consistent than those observed for crossbred heifer carcasses, in agreement with the results obtained by sensory and objective analyses. The data of *Bos indicus* carcasses submitted to PS and aged for 5 days were displaced towards the lower-right quadrant of the biplot, indicating positive and high values in the sensory analysis and negative and low SF and CL values. Inverse correlations were also observed in this multivariate space, for example, between SF and meat color (chroma and a *) and between sensory traits (tenderness, liking of flavor, juiciness and overall acceptance) and PL.

## 4. Discussion

Carcass suspension by the pelvic bone is a widely used method in Australia and in some European countries which is aimed at improving the quality traits of retail cuts from the high-value parts, especially sensory tenderness. Within this context, studies on cattle [13,15,28], small ruminants [29,30,31,32] and other species [33,34] have reported better meat tenderness in response to PS when compared to the traditional method (AS).

Other sensory quality attributes that are important for the consumer and are highly correlated with tenderness can also be influenced by the carcass-suspension method, such as liking of flavor, juiciness and overall acceptance [35]. The results obtained in studies on beef cattle and other species support the present findings, especially regarding sensory meat tenderness of Nellore bulls and crossbred heifers. Our results suggest that the PS method is more effective in improving meat quality traits of *Bos indicus* carcasses of bulls, which is a biological type with limited meat quality traits compared to crossbred heifers or steers [6,8]. In addition to the gender effects, the degree of tenderization caused by PS method is dictated by the genetic background of the animals, as reviewed [36].

The two biological models were divergent in terms of carcass traits, characterizing the distinction between the experimental groups. The mean hot carcass weight of 369.97 kg and dental maturity of 3.67 of Nellore animals demonstrate Brazilian market requirements that prioritize greater meat production per animal unit [37]. On the other hand, the market of beef with higher added value can only be supplied by products with the characteristics of crossbred cattle. With marbling scores three times higher than those of Nellore bulls and a younger age, these animals produce meat with better sensory quality [38].

### 4.1. Carcass and Meat Quality Traits

According to the MSA system, hanging carcasses by the pelvic bone during chilling or during the first 10 h of cold storage allows the shortening of the aging time of some cuts [18]. This hypothesis was confirmed in the present study for beef loins (LTL muscle), especially for Nellore carcasses and aged meat. Under these conditions, in addition to gains in the sensory quality variables of Nellore beef loins, interactions between the suspension method and aging were observed, which also contributed to a better instrumental meat quality in this group (lower SF and CL).

Considering that approximately 80% of CL refer to losses by evaporation, carcass-chilling methods that provide greater accumulation of water in the muscle tissue tend to reduce the effects of losses on the final sensory quality of meat during cooking [39]. However, the PS method does not affect the water-holding capacity—WHC (indirectly measured as CL and PL)—of meat in Nellore group, and opposite results can be found in the literature with *Bos indicus* bulls [13]. Similar results were reported by researchers working with young buffalo hung by PS method [40], whereby no changes in WHC of meat were found. Differences among studies may be due to factors such as genetic (species), gender status, aging period used and chemical composition of meat.

Similar results have also been reported by Juárez et al. [41] who showed that ~45% of the variations in the SF of beef were explained by the carcass-suspension method (AS or PS), aging (2-, 6-, 13-, 21- or 27-days post-mortem) and their interactions. Reductions in SF (~4.9 N) and CL (~2%) observed in samples of the present study have also been reported for beef samples of intact Ankole bulls derived from carcasses hung by the pelvic bone versus the traditional method [13].

The effect of the interaction between the suspension method and meat aging on the liking of flavor, juiciness and overall acceptance of Nellore beef, which was not observed for Brangus beef, is possibly due to the fact that meat from purebred *Bos indicus* bulls is more sensitive to the suspension technique than meat from crossbred Brangus young heifers. It is known that intense meat tenderization during the post-mortem period occurs earlier in animals with a higher percentage of the *Bos taurus* genotype [42], thus minimizing the early effects of technologies adopted in the industry.

Furthermore, Bresolin et al. [43] described possible causal relationships between hot carcass weight, ribeye area and SF in Nellore animals (*n* = 4414) with a weight and age similar to those of the present study, suggesting a significant relationship between meat tenderization and higher carcass weights. These results suggest greater effects of carcass chilling techniques on the meat quality of this biological type, considering both genetic/gender factors (Nellore bulls).

### 4.2. Meat Aging

Nian et al. [15] tested PS of Holstein steer versus bull carcasses aged for different periods (3, 7 or 14 days). These authors reported that PS was more consistent in reducing the SF of bull beef aged for 3 and 7 days when compared to the AS method. The differences between suspension methods on meat quality disappeared after 14 days of aging, as also observed in the present study at 15 days. It is therefore possible to infer that a longer aging time had a greater effect on sensory tenderness than the suspension method in both genetic groups. In general, studies on carcass suspension do not compare or combine this technique to others adopted in the industry such as wet aging; however, the MSA considers both factors to be determinant of final meat quality [18].

Taken together, these results suggest that the PS method may accelerate meat tenderization during the first days of aging, with a greater magnitude being observed for tougher meat (i.e., purebred *Bos indicus* animals and non-castrated). Such results agree with Watson et al. [18], who first reported that the effect of PS becomes quite small in samples subjected to extended aging. We also demonstrating that PS could be a fundamental post-mortem tool for the meat-packing industry, which seeks to accelerate the tenderization rate and to reduce the aging time of beef from Nellore bulls, the main biological type produced in feedlots in Brazil [1]. Within this context, PS is a low-cost technology that improves sensory and objective meat quality traits and can therefore add value to beef cuts.

Similar to the present study, Moran et al. [28] also reported benefits of PS on the sensory meat tenderness of Holstein bulls versus steers slaughtered at 19 and 24 months of age, respectively. The influence of PS on post-mortem aging rate was described in an MSA prediction model for beef palatability [14]. The authors explained that, in general, aging tends to even things out: when the meat palatability improves with age, it improves at a faster rate for the suspension method that is behind at 5 days. Thus, observation of the lack of effects of PS compared to AS on meat quality traits is more common at longer aging times (>14 days). Few studies are available in the literature that tested the effect of the PS method on bovine carcasses and evaluated longer aging times, especially in *Bos indicus* cattle. In the MSA model described by Watson et al. [14], the majority of the aging data were in the range of 7–21 days, with most of them being in the 11–14 days category.

### 4.3. Meat Quality Correlations

We found a strong correlation between different sensory scores (tenderness, juiciness, linking of flavor and overall acceptability), which agrees with MSA system [14,18]. Those authors also reported that these sensory variables generally show a very high correlation (r > 0.9), forming a new trait called ‘meat quality score’. The low phenotypic correlations between sensory and objective meat quality traits (r = −0.07 to −0.26) observed in the present study for Nellore bulls (Figure 1) and Brangus heifers (Figure 2) were expected since the sensory meat quality tests were conducted on untrained consumers.

According to the MSA researchers [18], objective measurements (such as SF) have the advantage of being relatively cheap and rather simplistic, one-dimensional measures of a complex set of interactions that occur when cooked meat is chewed and masticated in the mouth. Moreover, whilst SF is a useful indicator of sensory tenderness, it does not account for all the improvement in sensory scores when meat is aged, as observed in the present study using two aging times. This relates to the decreasing importance of the strength of the myofibrillar component of toughness with aging.

Although of low magnitude, the correlations between sensory tenderness and SF were inverse (*p* < 0.01) for both Nellore (r = −0.12) and Brangus beef (r = −0.26). Similar results (r = −0.23 to −0.28) have been reported in a study that evaluated the relationships between instrumental and sensory tenderness of three muscles from Belgian Blue and Norwegian Red cattle [44]. Factors such as the magnitude of the differences in tenderness between the experimental treatments (PS vs. AS), the use of an untrained panel of consumers in the sensory tests [18] and the small number of samples in the objective analysis may have affected the correlation results. On the other hand, SF was associated with higher CL in the two groups (r = 0.67 and 0.53, Figure 1 and Figure 2, respectively), results that agree with the data of sensory analysis.

In a previous study, researchers [45] used PCA to evaluate meat quality traits of Nellore cattle and found similar results, in which beef samples with higher SF and CL values (tough meat) commonly showed a lower backfat thickness and myofibril fragmentation index. Researchers investigated meat quality traits from male Holstein Friesian cattle using a multivariate approach and classified samples among tenderness clusters (tough, tender and very tender) in response to AS and PS meats aged for different days [28]. Similar to the present study, Moran et al. [28] also found that meat samples seemed to reach a greater degree of tenderness in a shorter aging period when the PS method was used. Additionally, the PCA results obtained for the meat quality traits of Nellore and Brangus animals agree with those reported by Lopes et al. [20], who applied multivariate techniques to evaluate meat quality traits of crossbred bulls. The meat color aspect, based on chromaticity of longissimus muscle, reveals greater influence of color variables on meat quality traits and corroborate the findings described in another multivariate study of Nellore heifers [46].

### 4.4. Limitations

Pelvic suspension is a well-accepted means of increasing tenderness by stretching the LTL muscle, which greatly changes the myofibrillar protein overlap and subsequently SF. At the same time, however, aging beef increases tenderness through increase proteolytic cleavage on intra-sarcomeric proteins that then allow for the disintegration of the myofibrillar structure. Additional biochemical (sarcomere length of LTL samples) or molecular studies (e.g., proteomic approach) are necessary to fully explain PS effects on muscle structure and, consequently, meat quality traits.

The main effects of PS or AS methods are slightly confounded on the biological groups used in the current study (combining genetic and gender factors; Nellore bulls versus Brangus heifers) because of the lack of overlap in carcass weight, marbling score and age. Consequently, several objective variables did not reach statistical significance, and interaction effects between carcass-suspension method and aging were divergent among groups (on sensory and objectives variables), which may be due to the small number of animals used for the meat quality assays. Taken together, these results suggest that a greater number of animals should be evaluated in the future. If a greater number of samples had been used, and a greater number of untrained consumers had participated in the sensory tests, perhaps the results could have been more consistent.

Further analyses could be undertaken to integrate the results into an overall model. It would be possible that increments in sensory eating quality (PS-AS) could be regressed against the sensory scores for AS sides. This would show that lower eating-quality carcasses showed a greater response to PS, an interaction that has been shown previously by other researchers [47,48]. Therefore, expressing the sensory data from the two biological groups eliminates (or at least minimizes) the difference between the two groups.

An economic evaluation could bring additional data to the present study. Although tenderstretch was proven to be effective in improving tenderness and other sensory variables, it was not widely adopted in Brazil and other countries due to the perceived inconvenience, extra costs and the lack of financial incentive for improved eating quality. When PS method is applied, there is a need to have one extra person on the chain to insert the hook through the pelvic bone, as well as one person to rehang the carcass by the AS after rigor mortis is complete.

## 5. Conclusions

Pelvic suspension of carcasses improved the beef loin quality of Nellore bulls and Brangus heifers, two very different biological types. The PS method allows a reduction in the aging time of Nellore beef from 15 to 5 days without compromising, or even favoring, sensory (tenderness, liking of flavor, juiciness and overall acceptance) and objective traits (SF and CL), demonstrating that animals with a higher genetic *Bos taurus* content have more tender meat after a shorter aging period. Pelvic suspension is therefore an efficient technology for significantly reducing the chilling period of Nellore carcasses (or with a high *Bos indicus* content and bulls), with a positive impact on the costs for the meat-packing industry. In Brangus heifers, the PS method had a positive effect on sensory meat quality traits such as tenderness, liking of flavor and overall acceptance, but did not influence objective quality traits.

## Figures and Tables

**Table 1:** Carcass measurements of feedlot-finished Nellore bulls (*n* = 10) and Brangus heifers (*n* = 10), whose half-carcasses were suspended after slaughter by the traditional Achilles tendon method (*n* = 20) or by the pelvic bone (*n* = 20).

Variables ^a^	Nellore Bulls (*Bos indicus*)		Brangus Heifers (*Bos taurus* × *Bos indicus*)		*p*-Value ^e^
	Mean	SD	Mean	SD	
Fatness score	2.50	0.55	3.50	0.84	0.034
Ribeye area, cm²	87.67	7.92	72.67	6.44	<0.01
Conformation score	3.17	0.75	3.17	0.41	ns
Hump height, mm	166.67	10.33	54.60	28.95	<0.01
Dental maturity ^b^	3.67	0.82	1.67	1.51	0.170
Backfat thickness ^c^, mm	4.17	1.83	8.00	2.61	0.014
Marbling score ^d^	108.33	20.41	301.67	53.07	<0.01
Ossification score	196.67	25.03	180.00	31.62	ns
Hot carcass weight, kg	369.97	8.96	264.87	14.15	<0.01
Final pH (48 h)	5.39	0.05	5.50	0.06	ns

^a^ According to Australian beef carcass evaluation—chiller assessment [17]. ^b^ Number of permanent incisor teeth. ^c^ At the 12th/13th rib interface. ^d^ According to Meat Standard Australia—MSA [18]. ^e^ ns = not significant.

**Table 2:** Sensory and objective meat quality traits of aged samples (5 or 15 days) from feedlot-finished Nellore bulls whose half-carcasses were suspended by the traditional Achilles tendon method (*n* = 20) or by the pelvic bone (*n* = 20).

	Carcass-Suspension Method										
	Achilles Tendon				Pelvic Suspension				*p*-Value ^a^		
	Aging Period										
Variables	5 d		15 d		5 d		15 d				
	Mean	SD	Mean	SD	Mean	SD	Mean	SD	S	A	S × A
Sensory											
Tenderness	61.02 ^a^	16.08	74.55 ^c^	13.96	69.85 ^b^	15.12	76.68 ^d^	14.08	0.003	<0.001	0.099
Liking of flavor	63.55 ^a^	14.23	75.08 ^b^	11.26	70.61 ^a^	14.96	72.00 ^b^	11.86	0.230	<0.001	0.005
Juiciness	62.80 ^a^	13.76	71.79 ^b^	12.72	70.02 ^a^	14.75	71.01 ^b^	14.22	0.085	0.002	0.017
Overall acceptance	64.45 ^a^	13.61	76.00 ^c^	12.02	71.79 ^b^	13.31	75.28 ^d^	11.71	0.058	<0.001	0.017
Objective											
Redness (a *)	13.42	0.60	12.52	1.15	13.92	0.75	13.05	0.65	ns	ns	ns
Yellowness (b *)	5.54	0.42	5.58	0.33	5.54	0.42	5.61	0.43	ns	ns	ns
Lightness (L *)	30.34	1.75	31.03	1.76	30.82	1.81	31.16	2.53	ns	ns	ns
Hue	21.33	1.26	24.16	2.77	21.70	0.78	23.30	2.06	ns	ns	ns
Chroma	14.41	0.65	13.72	0.10	14.98	0.83	14.21	0.59	ns	ns	ns
Meat pH	5.46	0.05	5.53	0.03	5.49	0.01	5.53	0.04	ns	ns	ns
Drip loss, %	6.01	0.01	6.70	0.01	5.10	1.5	5.8	1.5	ns	ns	ns
Evaporation loss, %	25.90	2.01	24.20	2.10	24.90	2.91	21.90	3.10	ns	ns	ns
Cooking loss, %	32.01 ^a^	3.01	30.90 ^b^	1.61	30.10 ^b^	3.01	27.70 ^c^	3.60	0.042	0.167	0.578
Purge loss, %	3.40	1.60	2.80	1.10	3.40	1.60	2.60	1.10	ns	ns	ns
Shear force, N	50.40 ^a^	8.04	48.34 ^b^	7.35	44.62 ^c^	6.96	42.95 ^c^	5.00	0.061	0.521	0.952

S, suspension method; A, aging period; S × A, interaction; ns = not significant. Means with different superscript letters in the same row differ (*p* < 0.05, Tukey test).

**Table 3:** Sensory and objective meat quality traits of aged samples (5 or 15 days) from feedlot-finished Brangus heifers whose half-carcasses were suspended by the traditional Achilles tendon method (*n* = 20) or by the pelvic bone (*n* = 20).

	Carcass-Suspension Method										
	Achilles Tendon				Pelvic Suspension				*p*-Value ^a^		
	Aging Period										
Variables	5 d		15 d		5 d		15 d				
	Mean	SD	Mean	SD	Mean	SD	Mean	SD	S	A	S × A
Sensory											
Tenderness	68.15 ^a^	20.43	70.30 ^a^	15.11	76.75 ^b^	16.91	75.56 ^b^	11.13	<0.001	0.936	0.372
Liking of flavor	71.95 ^a^	16.06	67.80 ^c^	10.95	76.48 ^b^	15.62	69.83 ^c^	12.61	0.057	0.003	0.416
Juiciness	69.55	14.98	68.02	12.58	72.81	16.20	69.02	12.64	ns	ns	ns
Overall acceptance	73.38 ^a^	15.70	71.31 ^a^	11.96	77.96 ^b^	14.98	73.03 ^b^	11.93	0.063	0.083	0.445
Objective											
Redness (a *)	13.48	1.29	12.13	0.70	14.28	1.13	12.77	1.87	ns	ns	ns
Yellowness (b *)	5.53	0.83	5.50	0.46	5.98	0.86	5.91	0.60	ns	ns	ns
Lightness (L *)	32.13	4.67	33.68	4.25	33.80	4.19	33.52	3.93	ns	ns	ns
Hue	22.26	1.94	24.42	2.05	22.65	2.27	25.05	3.25	ns	ns	ns
Chroma	14.58	1.45	13.32	0.69	15.49	1.27	14.09	1.79	ns	ns	ns
Meat pH	5.47	0.03	5.51	0.06	5.52	0.04	5.50	0.05	ns	ns	ns
Drip loss, %	6.60	1.90	5.80	1.40	6.40	0.70	6.01	1.80	ns	ns	ns
Evaporation loss, %	21.40	4.01	19.40	2.60	20.70	2.40	21.20	1.60	ns	ns	ns
Cooking loss, %	28.00	4.70	25.10	3.10	27.00	2.80	27.20	2.20	ns	ns	ns
Purge loss, %	1.20	0.60	1.90	1.50	2.10	1.70	2.10	2.80	ns	ns	ns
Shear force, N	35.89	9.22	31.87	5.58	35.99	7.55	32.95	6.18	ns	ns	ns

S, suspension method; A, aging period; S × A, interaction; ns = not significant. Means with different superscript letters in the same row differ (*p* < 0.05, Tukey test).

## Data Availability

All relevant data are within the paper.

## Supplementary Materials

The following supporting information can be downloaded at: https://www.mdpi.com/article/10.3390/foods12050930/s1, Table S1. Results of principal component analysis for the first five principal components (PC). Table S2. Coefficients of the eingenvectors (loadings) for the first four principal components for meat quality traits of Nellore bulls. Table S3. Coefficients of the eigenvectors (loadings) for the first four principal components for meat quality traits of Brangus heifers. Figure S1. Small boxes (Brazil Beef Quality Ltd., Piracicaba, São Paulo, Brazil) with divisions used in sensory assays.
