# Supplementary material for: Bos indicus Carcasses Suspended by the Pelvic Bone Require a Shorter Aging Time to Meet Consumer Expectations Regarding Meat Quality
Source: Foods. 2023 Feb 22;12(5):930. doi: 10.3390/foods12050930 (PMC10000897; doi:10.3390/foods12050930)
Supplement: Supplementary file 1 [file foods-12-00930-s001.zip › foods-2159435-supplementary.pdf]

Table S1. Results of principal component analysis for the first five principal components (PC).

	Eigenvalues	Variance percentage	Cumulative variance percentage
Nellore bulls			
PC1	3.89	25.90	25.90
PC2	3.37	22.44	48.34
PC3	2.36	15.76	64.10
PC4	1.53	10.22	74.32
PC5	1.38	9.17	83.49
Brangus heifers			
PC1	3.71	24.73	24.73
PC2	2.74	18.26	42.99
PC3	2.69	17.94	60.93
PC4	2.31	15.37	76.29
PC5	1.16	7.72	84.01

Table S2. Coefficients of the eingenvectors (loadings) for the first four principal components for meat quality traits of Nellore bulls.

	PC1	PC2	PC3	PC4
Variable †	Tenderness	Meat color 1	Meat color 2	Cooking losses
TE	0.64	0.49	0.33	0.05
JU	0.60	0.48	0.45	0.00
FL	0.60	0.52	0.43	0.00
OA	0.65	0.51	0.44	0.02
L	0.23	-0.19	0.09	0.27
a	0.43	-0.73	0.14	0.42
b	0.31	0.31	-0.66	0.41
CH	0.47	-0.65	0.01	0.48
HUE	-0.07	0.74	-0.58	0.00
pH	0.56	-0.01	-0.33	0.06
SF	-0.71	0.50	0.24	0.07
CL	-0.65	0.28	0.34	0.60
DL	-0.27	0.58	-0.31	0.37
EL	-0.58	0.02	0.54	0.47
PL	-0.33	-0.24	0.45	-0.40

† TE = sensory tenderness; JU = juiciness; FL = flavor; OA = overall acceptance; L = lightness; a = redness; b = yellowness; CH = chromaticity; HUE = real color; pH = meat pH; SF = shear force; CL = total cooking losses; DL = drip loss; EL = evaporation loss; PL = purge loss.

Table S3. Coefficients of the eigenvectors (loadings) for the first four principal components for meat quality traits of Brangus heifers.

Variable †	PC1<br>Tenderness	PC2<br>Meat color 1	PC3<br>Cooking losses	PC4<br>Meat color 2
TE	0.80	0.23	0.09	-0.25
JU	0.78	0.27	0.12	-0.37
FL	0.81	0.27	0.14	-0.31
OA	0.83	0.29	0.12	-0.33
L	0.08	0.53	0.42	0.62
a	0.41	-0.68	0.43	0.32
b	0.42	0.11	0.25	0.85
CH	0.45	-0.59	0.43	0.45
HUE	0.05	0.73	-0.17	0.55
pH	0.19	-0.69	0.06	-0.15
SF	-0.49	0.38	0.53	-0.15
CL	-0.32	0.18	0.82	-0.23
DL	-0.01	-0.13	0.60	-0.07
EL	-0.39	0.28	0.68	-0.24
PL	-0.19	-0.26	0.46	-0.18

† TE = sensory tenderness; JU = juiciness; FL = flavor; OA = overall acceptance; L = lightness; a = redness; b = yellowness; CH = chromaticity; HUE = real color; pH = meat pH; SF = shear force; CL = total cooking losses; DL = drip loss; EL = evaporation loss; PL = purge loss.

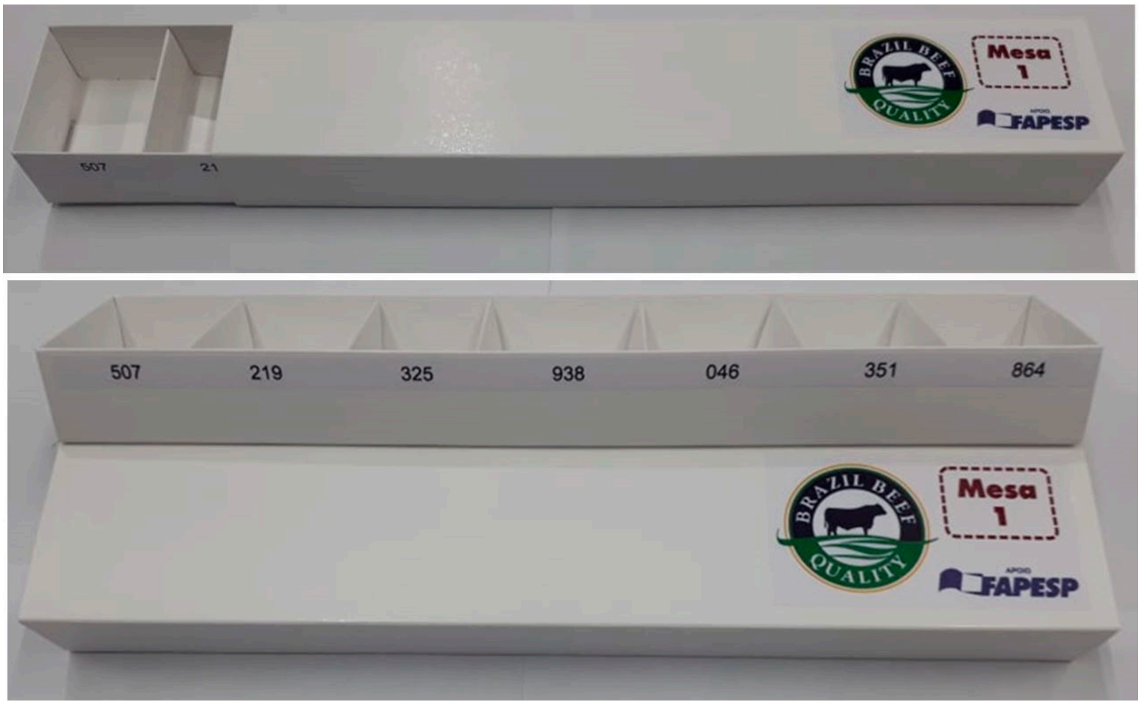

Figure S1. Small boxes (Brazil Beef Quality Ltd., Piracicaba, São Paulo, Brazil) with divisions used in sensory assays.
